# Persons suffering from severe and persistent mental illness with a persistent death wish: A cross-sectional study of the Reakiro care model

**DOI:** 10.1371/journal.pmen.0000486

**Published:** 2025-12-03

**Authors:** Thijs Vanhie, Sofie Verdegem, Patrick Onghena, Joris Vandenberghe

**Affiliations:** 1 Reakiro, University Psychiatric Centre, KU Leuven, Leuven, Belgium; 2 Methods, Individual and Cultural Differences, Affect and Social Behavior (MICAS), Faculty of Psychology and Educational Sciences, KU Leuven, Leuven, Belgium; 3 Department of Neurosciences, University Hospitals and University Psychiatric Centre, KU Leuven, Leuven, Belgium; UTHSC: The University of Texas Health Science Center at Houston, UNITED STATES OF AMERICA

## Abstract

Persons who suffer from unbearable psychiatric illness and a persistent death wish (chronic suicidality and/or a psychiatric euthanasia request) are an understudied subgroup of persons with Severe and Persistent Mental Illness (SPMI). They receive tailored care in Reakiro, a Belgian drop-in, care and expertise centre, where persons can enrol in existential counselling or peer support groups. Suicidology and psychiatric euthanasia literature identify several risk and protective factors that play a central role in the process of death wishes: meaning, hope, empowerment, suicidal ideation, existential anxiety, and psychosocial dysfunctioning. The aim of the study is to describe these central factors in this subgroup of persons attending Reakiro and compare them with other patient samples worldwide in order to position this subgroup in terms of severity of suffering. Another aim was to evaluate how the Reakiro care was experienced by the users. The Beck Scale for Suicide Ideation (M = 21.78, SD = 8.48), Netherlands Empowerment List (M = 113.39, SD = 20.42), Herth Hope Index (M = 24.15; SD = 5.40), Meaning In Life Measurement (M = 5.39, SD = 1.50), Existential Concerns Questionnaire (M = 62.13, SD = 12.11), and Outcome Questionnaire-45 (M = 98.93, SD = 20.50) showed very high suicidal ideation and severe suffering across all domains. The Reakiro sample scored almost exclusively worse on all factors compared with 20 samples worldwide. Users perceived Reakiro care as helpful, with individual counseling services as most helpful. Reakiro care seems to be a promising model appealing to this subgroup of persons with SPMI and a persistent death wish. These results should be interpreted with caution, as the cross-sectional design has several limitations. Replication or falsification with forthcoming longitudinal data is needed. Clinical implications of the results are discussed.

## Introduction

People who report unbearable suffering and a persistent death wish due to psychiatric illness are viewed as a subgroup of patients with Severe and Persistent Mental Illness (SPMI) [1,2]. They suffer from at least one, but usually multiple psychiatric disorders, in a chronic course (several years of treatment and prolonged illness), resulting in severe limitations in their daily psychosocial functioning [3,4].

Their struggle with life and death and diminished perspective on the alleviation of their suffering can result in the development of a persistent death wish: a wish to die, usually present or recurring for several years. Hospitalized psychiatric patients with SPMI are especially at risk for suicide as studies report elevated incidence up to 9.8% [5]. A persistent wish to die is to be distinguished from acute suicidal tendency, which is described as a sudden state of immediacy for suicide, impulsive suicidal thoughts and actions, triggered by stressful events, whereas chronic suicidal tendency is characterized by a persistent ideation for several months or years and is associated with compulsive cognitive processes [6–8]. In some cases the persistent death wish might even be perceived as rational, which means not stemming from psychiatric illness, but from a well-considered, deliberate thought process [9,10]. In an increasing number of countries (e.g., the Netherlands, Belgium, Luxembourg, Switzerland, Austria, Spain, Portugal, Slovenia,…) legislations regulate such deliberation processes between physician and patient. Most common terms used are Medical Assistance in Dying (MAID), Physician-Assisted Suicide (PAS) or Dying (PAD), when the lethal drugs are self-administered, and Psychiatric Euthanasia (PE), when the physician administers the lethal drugs [11]. This article is written in the Belgian context, so we will further use the term PE [12]. We acknowledge that the ethical and legal debates should continue in order to improve nuanced, meaningful dialogue and will further on focus the scope of this article on the health care for persons with a persistent death wish.

These different types of suicidality and euthanasia can be seen as different, not mutually exclusive expression forms of a death wish. Research remains scarce on whether suicidal persons and persons requesting PE are distinct subgroups or one group with merely a different expression of their death wish. Nicolini et al. [13] stress the importance of studying both groups together, because of considerable overlap in characteristics (such as a gender gap). A systematic review of six retrospective studies found positive, but non-significant associations between PE and suicide rates and thus concluded that PE requests do not decrease the rate of non-assisted suicide in this population, which implies that suicide risk remains high in the population of PE requesters [14]. These studies underline the interconnectedness of both phenomena.

Reakiro, a Belgian pilot project and drop-in, care and expertise centre for people who request PE or suffer from a persistent death wish, seems to be able to reach this population with its newly developed care model [15]. This model is based on existential, recovery-oriented, palliative and presence theories and aims at specialized mental healthcare for this vulnerable patient group. The main aim of the Reakiro research project is to explore what good practice could possibly look like for this specific group of SPMI patients. Box 1 succinctly delineates the Reakiro care setting and its aims. The interested reader can find more in-depth information on Reakiro care in Vanhie et al. [15].

**Box 1. Reakiro setting**

Reakiro is a drop-in, care and expertise centre in Belgium for people with SPMI and a persistent death wish. Patients can enrol in existential counselling and peer-support groups based on meaning-centred therapy and recovery-oriented practice, exploring meaning in life, hope, empowerment and existential anxiety [15]. Autonomy, connectedness, dignity, harm reduction and shared decision-making in continuing treatment and/or starting the PE procedure are central values of the project. The aim of Reakiro is to be a safe, non-judgemental, holding space where persons can explore their life-and-death ambivalence and the layered meanings their death wish contains in order to increase awareness, meaning making and informed, deliberate decision-making towards either life or death. The relationship with the Reakiro caregiver is seen as the most crucial protective factor. Reakiro is a care facility that adheres to Flemish suicide prevention guidelines and works together with other organizations where treatment is continued and where the PE requests are evaluated (cf. the two-track approach). Persons in Reakiro care have an actively treating GP, psychiatrist, and psychologist as a care network. If acute suicidal ideations or actions arise, Reakiro caregivers will reach out and aim for strong connection in the therapeutic contacts and collaborate intensively with the care network, including crisis care.

PE literature and suicidology offer insight into which underlying factors are of central concern. Several studies describe PE requesters as suffering severely on multiple interwoven dimensions: biological, psychological, social and existential, with existential suffering being the primary reason for requesting PE [16–19]. Comorbidity of two or more DSM-V vulnerabilities is common in this population in Belgium, with mood and personality disorders being the most prevalent [15,18,20]. A persistent death wish may be understood as a long-term adaptation and coping style to chronic mood disorders and/or personality disorders: the need to feel in control over one’s suffering [7]. Based on these epidemiological studies we can fairly assume that suicidal ideation and behaviour is a common feature of the population of PE requesters, although not all PE requesters retain suicide as a viable option as it is often perceived as a less dignified way of dying than PE [16,18].

Physicians assessing the death wish in PE requesters try to differentiate between impulsive suicidality, chronic suicidality and a rational death wish, with the latter being perceived as more acceptable as a reason for PE (yet still highly controversial and debated) [21]. Some physicians conclude that the main difference between chronic suicidality and a rational death wish would be that the former stems from psychiatric illness and would therefore require treatment, as the latter is perceived by the physician as a more logical and deliberate reflective process separate from any concurring illness [21]. Other physicians argue that these are not always easy to separate and can possibly co-occur in persons that are deemed decision-competent and able to request PE [22]. The prevalence of suicidal ideation and active PE requests in the population of people who suffer from unbearable psychiatric illness and a persistent death wish will be further explored in this study in order to add to the empirical body of knowledge on these expressions of death wishes [16,18].

Suicidology identifies different risk factors as key targets for prevention and intervention of suicide such as suicidal ideation, number of prior attempts, mental illness (e.g., depression) and feelings of hopelessness and meaninglessness [23–25]. These are established and form a solid ground for researching our population of people with a persistent death wish. However, as suicide rates remain high, this disease-oriented focus on psychiatric illness might be too partial to capture the full scope of complexity. Emerging research suggests that palliative psychiatry, existential, recovery-oriented and positive psychology approaches could possibly enhance the care for this population [24,26–29]. Trachsel and colleagues [29] describe palliative psychiatry as: “Improving quality of life of patients and their families in facing problems associated with life threatening severe and persistent mental illness through the prevention and relief of suffering by means of a timely assessment and treatment of associated physical, mental, social and spiritual needs.” (p.3) These approaches target protective factors and experiential growth processes such as meaning in life, hope and empowerment by attuning to concerns and needs of patients and their families and by focusing on harm reduction and reducing possibly burdensome psychiatric interventions. In convergence with contemporary research on suicide, both risk- and protective factors will be included in this study’s exploration. Critchfield and Harvell-Bowman [30] suggest another understudied factor at play in the formation of chronic suicidality: existential anxiety. Tillich [31] describes existential anxiety as an anxiety on existence itself, usually expressed as death anxiety or as an anxiety triggered by the realization that life is inherently meaningless. Persons would normally use anxiety-reducing coping mechanisms such as meaning making, attachment and connectedness to survive and manage their death anxiety [32,33]. However, SPMI patients with a persistent death wish might find solace in death as a way out of their overwhelming (existential) anxieties and suffering. Based on abovementioned literature we could expect to find lower levels of hope, meaning and empowerment and a heightened existential anxiety, suicidal ideation and psychosocial dysfunctioning in our sample of persons with SPMI and a persistent death wish.

## Aim

This cross-sectional study has two aims: firstly, we will explore descriptively how these persons with SPMI relate to several key factors in the persistent death wish process: their life and death wish, the severity of their death wish, meaning in life, hope, empowerment, existential anxiety, and a general outcome measure of their psychosocial dysfunctioning. We will check if and how these variables are related and compare them with other patient samples worldwide in order to empirically position this subgroup in terms of severity of suffering. It is of specific interest to examine if this sample forms a homogenous whole or is built up from specific subgroups who differ in their profile and therefore maybe in their care needs and trajectories. Secondly, we also want to explore how the users experience and evaluate the Reakiro care.

## Method

### Ethics statement

Approval of the Ethics Committee of University Hospitals KU Leuven was obtained (S64625). Formal written informed consents were obtained.

### Participants and setting

**Fig 1:**
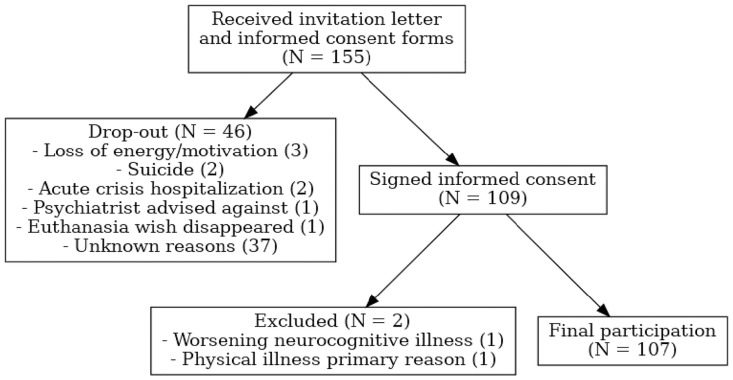
Participant flow diagram.

Persons were eligible to participate in the study if they had been seen on intake at least once in the Reakiro centre and if they verbally expressed a persistent death wish (chronic suicidality and/or wish for PE) with their Reakiro counsellor. Reakiro users were recruited from two Reakiro centres in Leuven and Bruges and were simultaneously enrolled in several in- and outpatient care programs across Flanders, ensuring recruitment from diverse demographical and clinical backgrounds and reducing possible selection bias. First notification of the study was given by an invitation letter handed over by the Reakiro counsellor at intake (see Fig 1 for a participant flow diagram). If persons expressed interest in participation, they received the informed consent and had a week to decide on participation. All persons in Reakiro were eligible for participation, unless the Reakiro counsellors assessed the decision-making capacity, executive functioning and/or orientation in time and space to be impaired (e.g., acute states of severe psychosis or dissociation). After one week of deliberation, a walkthrough of the informed consent and questionnaires was organised by phone call or live in Reakiro. After obtaining written informed consent, the participants received the questionnaires through the Research Electronic Data Capture system (REDCap), used for survey distribution, data collection, protection and management [34]. Participants were recruited from 01/02/2022 until 31/06/2024. SPSS was used for data-analysis.

### Questionnaires

Questionnaires that were unavailable in Dutch were translated by two doctoral researchers using a back translation method. An English native speaker and doctoral researcher, independent from the research group, translated this version back to English, which allowed the researchers to find discrepancies in translation and find consensus on adequate translation without losing original meaning.

*The Beck Scale for Suicide Ideation (BSSI)* measures suicidal ideation [35]. The German version shows a Cronbach’s Alpha of 0.94. Construct validity was satisfactory. It contains 21 statement groups, each one consisting of three sentences representing an increasing intensity of suicidal ideation on a 3-point scale (0–2).

*The Herth Hope Index *(HHI)** is a measurement of hope. It has been tested and deemed reliable and valid in a sample of Dutch patients with severe and persistent mental illness (SPMI), reporting a Cronbach’s Alpha of 0.84 and test-retest reliability of 0.79 [36]. It contains 12 Likert scale items with scores ranging from 1 (strongly disagree) to 4 (strongly agree).

*The Meaning In Life Measure *(MILM)** is a measurement of meaning. It includes items related to mattering, goal, coherence and reflectivity [37]. A high internal consistency of 0.85 and test-retest reliability of 0.87 are reported. It is an eight-item questionnaire with a scoring range from 1 (extremely disagree) to 9 (extremely agree) per item. Total score is the mean of the 8 items.

*The Netherlands Empowerment List *(NEL)** measures empowerment [38]. Cronbach’s Alpha is 0.94. Similar to the HHI, this questionnaire was constructed and validated in a sample of Dutch patients with SPMI. It is a 40-item self-report questionnaire with 5-point Likert scales. Validity, reproducibility (intraclass correlation = .79) and responsiveness are demonstrated to be good.

*The Existential Concerns Questionnaire (ECQ)* measures existential anxiety, covering five domains of existential concerns: death, meaninglessness, guilt, social isolation and identity [39]. Cronbach’s Alpha is 0.91 in the clinical sample. Psychometric analysis showed a unidimensional scale and a high interrelatedness of the five existential domains. It contains 22 items, scored on a 5-point Likert scale (never – always).

The Dutch *Outcome Questionnaire (OQ-45)* is used as a general outcome measure [40]. This instrument is a widespread tool to assess therapeutic change and clinical scores of psychosocial distress and dysfunctioning. 45 items are scored on a 5-point scale from never (0) to almost always (4). The Dutch version reports psychometric equivalence for internal consistency (Cronbach’s Alpha = 0.93), test-retest reliability (r = 0.79), and validity to the American original version [40]. Cut-off scores were calibrated for the Dutch population in order to retain a satisfactory criterium validity.

Two specific *Reakiro questionnaires* were added to grasp the context of the drop-in centre. Sixteen items assess the wish to die, the wish to live, the impact of the Reakiro care model on their life-death decision making, the position in the euthanasia trajectory and helpful factors of the Reakiro care model. These Reakiro questionnaires are psychometrically unvalidated instruments. Questionnaires are available at the Open Science Framework (https://osf.io/c9u3j/).

### Procedure

Descriptive statistics were used to analyse the demographical and questionnaire data and assess how users rated the Reakiro care model. Relation of the life and death wish was visualized with a stacked bar diagram to explore and differentiate this relationship. Boxplots with visualisation of individual data points were used to display the overall sample, assess heterogeneity of the sample, and search for possible subgroupings in the different assessed variables. Outliers that were identified with boxplots, were kept in the analysis as these could yield important information, e.g., absence of suicidal ideation in some cases. The Primo search engine (Ex Libris Knowledge Center) was used to find data samples collected with the same questionnaires as used in this study. SPMI and the names of the questionnaires were used as keywords in the queries. The goal was to compare other clinical and SPMI samples to our Reakiro sample in order to empirically indicate where the Reakiro sample could be positioned in terms of severity of SPMI and suffering on the different variables measured. Spearman’s rho correlations were calculated to assess the relationships between different variables and to indicate how time spent in Reakiro and mental healthcare would be associated with these variables. Normality was not achieved for suicidal ideation, hope and meaning as tested with Shapiro-Wilks test and q-q-plots. Pairwise deletion was used. Benjamini Hochberg correction was applied on p values.

A post-hoc power analysis with G*Power (N = 99, α = .05, two-tailed) indicated that the sample size was adequate to detect medium and large correlations (r = .3, power = .86; r = .5, power = .99), but not large enough to detect small correlations (r = .1, power = .17). A sensitivity analysis (N = 99, α = .05, two-tailed) indicated a minimal detectable correlation of.28 at 80% power. The critical correlation for significance was r = .20.

## Results

31.8% of participants identified as male, 64.5% as female, and 3.7% as gender diverse (N = 107). 10.3% were young adults (18–24 years), 84.1% were adults (25–64 years), and 5.6% were elderly (65 + years). Comorbidity was common in this sample, with mood, personality and trauma-related disorders again being the most common diagnoses. These numbers are similar to previous Belgian epidemiological studies, confirming adequate representation of the broader population of persons with SPMI and a persisting death wish (Vanhie et al., 2025). Self-reported time participating in Reakiro care averaged 5.28 months (SD = 7.32) at the timing of the study, with a maximum reported time of three years. 58.9% of the sample had only just started their care trajectory in Reakiro (≤2 months of enrolment). Almost all participants (except eight) indicated to be in the care of mental health services for three or more years. Three persons reported zero time in mental healthcare services. A subgroup (35 persons) reported being in mental healthcare for two or more decennia. The self-reported time enrolled in mental health services averaged 14.25 years (SD = 10.59).

Table 1 clarifies missing data for each variable. One person filled in the demographical data and then stopped for unknown reasons. Five persons left the field to report diagnoses blank, due to being unaware of their diagnoses or due to still being on a waiting list for formal diagnostics in their respective care facilities. Missing data for the Reakiro questionnaires varied between items from one to three persons. Throughout the questionnaire battery participation dropped to 99, possibly due to the length of the total form. Some participants reported quitting due to attention or executive functioning problems.

**Table 1:** Missing data by variable in order of assessment.

Variable	Total N	Missing (N)	Missing (%)
Age, gender, enrolment in Reakiro care	107	0	0%
Comorbidity	102	5	4.7%
Enrolment in mental healthcare	104	3	2.8%
Reakiro questionnaires	104-106	1-3	0.9-2.8%
SSI	106	1	0.9%
NEL	100	7	6.5%
HHI, MILM, ECQ, OQ-45	99	8	7.4%

A majority of participants reported not being in the formal euthanasia procedure (63.5%), approximately a quarter of the participants was enrolled in the formal euthanasia procedure (26.2%; Table 2). A minority reported a persisting death wish, but no wish for euthanasia (14.0%) and another minority group reported not holding any current death wish (5.6%).

**Table 2:** Position in euthanasia trajectory.

	Frequency (%)
Persisting death wish, no wish for euthanasia	15 (14.0)
Persisting death wish, a wish for euthanasia, but not enrolled in the formal procedure	47 (43.9)
Persisting death wish, enrolled in the formal euthanasia procedure	28 (26.2)
No current death wish, nor a current wish for euthanasia	6 (5.6)
Total	97 (90.7)
Missing	10 (9.3)

**Fig 2:**
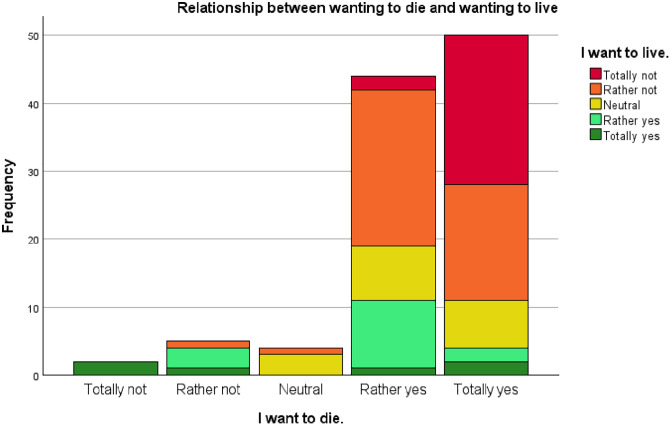
Relationship between wanting to die and wanting to live.

An indication of the wish to die and the wish to live was measured in the Reakiro questionnaire with two items on a five point Likert scale: “I want to die.” (M = 3.29, SD = 0.89) and “I want to live.” (M = 1.40, SD = 1.16). A stacked bar diagram displays how both wishes relate to each other (Fig 2). The wish to die is very strong in this sample. However, less than half of the participants who answered ‘totally yes’ for their death wish, also answered ‘totally no’ for their life wish. Some individuals even indicated that they would rather not live and rather not die or felt neutral towards living or dying. Several individuals indicated a wish to live, sometimes accompanied with a wish to die, but sometimes this wish to die was more absent.

Perceived impact of the Reakiro care facilities on choice for life and death was measured with a slide bar ranging zero (strengthened wish) to 100 (weakened wish). On average participants perceived a minor strengthening impact on the choice for life (M = 46.36, SD = 13.41) and almost no impact on the choice for death (M = 49.75, SD = 15.11). Looking at individual cases, 22 persons indicated a small to large strengthening of their life wish and four persons indicated a small weakening of their life wish. Six persons indicated a small and one person a large strengthening of their choice for death and 23 persons indicated small to large weakening of their choice for death. 55 and 57 persons reported no impact on their wish to live or die respectively.

Five items on five-point Likert scales (1 = totally not; 5 = totally yes) assessed different helping experiences in the care model of Reakiro (Table 3). People could also tick a ‘not applicable’ box, if they did not participate in a particular part of the Reakiro care. A sixth item (1 = Yes, 2 = No) assessed whether Reakiro had added care value next to other care facilities where the persons were participating. All Reakiro care facilities were generally experienced as helping by their users. Individual counselling services were reported as most helpful. On average participants reported to find additional care at Reakiro that they did not find in other care facilities (M = 1.08, SD = 0.27).

**Table 3:** Experiences of care in Reakiro.

	N	M	SD
I experience the care facilities at Reakiro as helping.	106	4.14	0.76
The peer support group at Reakiro is helping me.	42	3.64	0.96
The individual care at Reakiro is helping me.	104	4.22	0.65
The opportunity to walk in without an appointment is helping me.	61	3.85	0.89
The opportunity to contribute to the care of Reakiro is helping me.	69	3.59	0.94
I find care at Reakiro that I did not find at other care facilities.	106	1.08	0.27

The BSSI results showed that 28.30% of participants in Reakiro never attempted suicide, 17.90% attempted suicide once, and 53.80% attempted suicide twice or more. Reakiro participants scored very high for suicidal ideation (M = 21.78, SD = 8.48; Table 4), compared to samples of persons admitted to emergency services after suicide attempts or compared to other vulnerable, minority groups such as transgender persons (M = 10.21-17.5, SD = 6.73-9.59) [41–44].

**Table 4:** Descriptive analysis psychometrically validated questionnaires.

	N	M	SD
BSSI	106	21.78	8.48
DES	100	113.39	20.42
HHI	99	24.15	5.40
MILM	99	5.39	1.50
MILM-E	99	4.91	1.43
MILM-R	99	5.87	2.10
ECQ	99	62.13	12.11
OQ-45	99	98.93	20.50

The NEL total score showed a lowered rate of empowerment in the Reakiro sample (M = 113.39, SD = 20.42), when comparing with a Spanish sample of mental health care users suffering from mood, personality and psychotic disorders (M = 138.32, SD = 25.88) [45]. Other studies with Belgian and Dutch persons with SPMI used a mean item score to measure empowerment. The Reakiro mean item score for empowerment (M = 2.84, SD = 0.50) was still lower than those of the other studies (M = 3.32-3.55, SD = 0.51-0.67) [46,47]. Only one study (with depressed persons) found a lower empowerment score (M = 1.99, SD = 0.5) [48].

The HHI total score showed a lowered rate of hope (M = 24.15; SD = 5.40), when comparing with other European chronically and terminally ill patient groups (cancer, diabetes, cystic fibrosis) who had HHI average scores ranging from 34.00 to 41.63 [49–52]. When comparing with three other samples of persons with SPMI (mood, psychotic and, personality disorders), the Reakiro sample still reported the lowest levels of hope (M = 27.00-35.07) [53–55].

The MILM total score showed a lowered rate of meaning (M = 5.39, SD = 1.50), when comparing with Belgian and US student and general population samples (M = 6.94-7.50, SD = 1.07-1.39) [37,56]. The MILM-E subscale showed an even lower experience of meaning (M = 4.91, SD = 1.43), when comparing with the US sample (M = 7.13-7.45, SD = 1.34-1.51) [37].

The ECQ total score showed a higher level of existential anxiety in the Reakiro sample (M = 62.13, SD = 12.11), compared to a Dutch clinical sample of persons visiting an outpatient treatment department for anxiety and mood disorders (M = 58.34, SD = 13.75) [39].

The OQ-45 total score showed very high levels of clinical dysfunctioning (M = 98.93, SD = 20.50), compared to several US and Dutch clinical samples (M = 67.4-92.3, SD = 20.2-22) [40,57]. Most of the Reakiro participants scored above the Dutch cut-off score (64.7) for comparison with a full-time inpatient sample [57].

**Fig 3:**
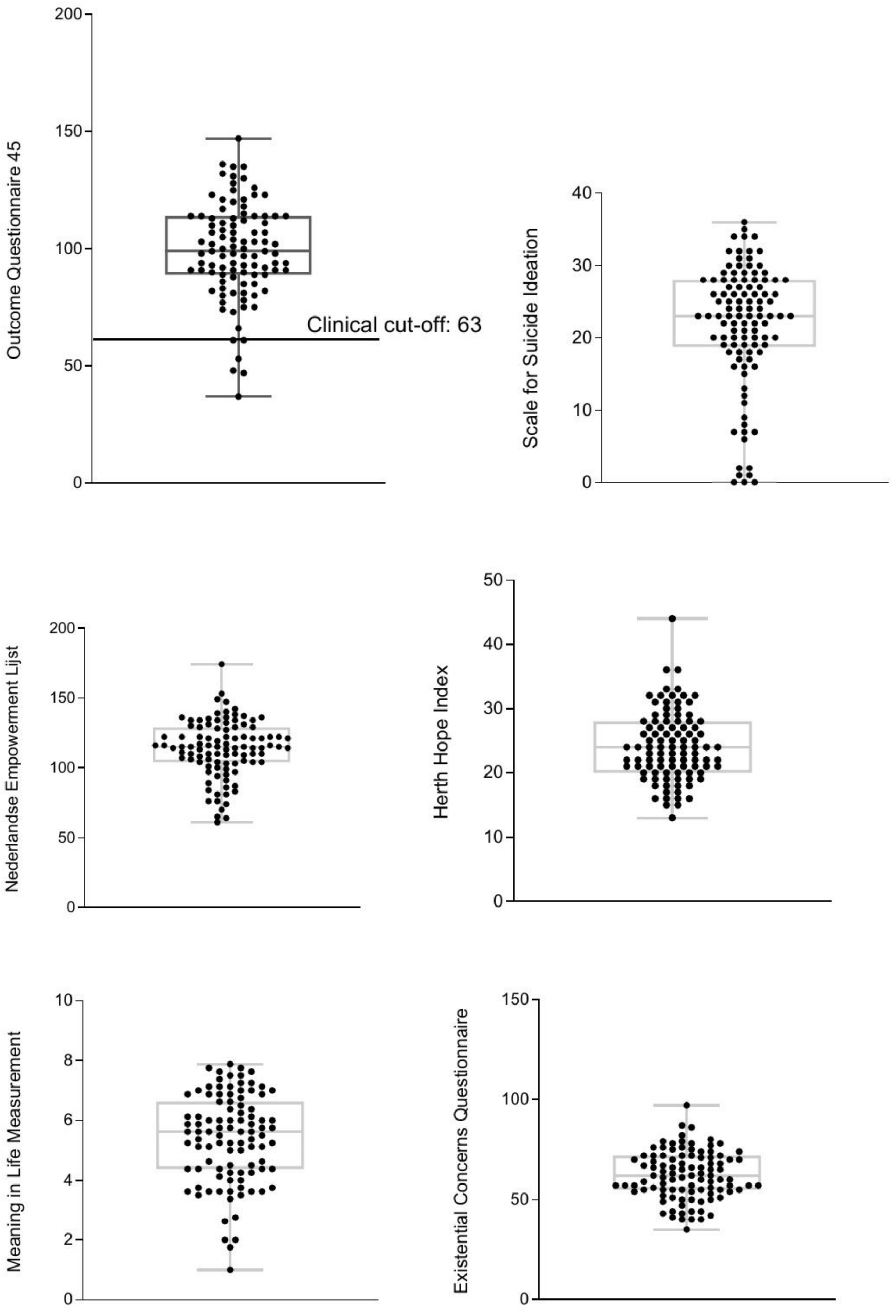
Boxplots of key factors.

Boxplots with individual data points were made to check for homogeneity and to visualize individual differences in the sample (Fig 3). Four persons did not meet the clinical cut-off for psychosocial dysfunctioning on the OQ-45. Seven persons did not report any suicidal ideation and nine persons scored on the lower end of suicidal ideation. Empowerment, hope and existential anxiety show a more homogenous sample, whilst suicidal ideation and meaning in life show more heterogeneity in the sample. However, the variance of the Reakiro sample seems to be within the same ranges as those of other study samples (see above).

Spearman correlations show significant associations between several key factors (Table 5). Time spent in mental healthcare showed a moderate correlation with time spent in Reakiro care (*r* = .251, *p* = .027) and with a lower suicidal ideation (*r* = -.246, *p* = .030). Time enrolled in Reakiro care did not correlate significantly with any of the other factors. Suicidal ideation was associated with lower levels of empowerment (*r* = -.254, *p* = .026), hope (*r* = -.261, *p* = .022), and meaning in life (*r* = -.334, *p* = .002), and higher levels of existential anxiety (*r* = .229, *p* = .044) and psychosocial dysfunctioning (*r* = .356, *p* = .001). Empowerment, hope and meaning experience showed strong, positive associations with each other, and negative associations with existential anxiety and psychosocial dysfunctioning. Negative correlations between meaning (experience) and existential anxiety were similar compared to other Belgian samples [56,58].

**Table 5:** Spearman’s rho correlation matrix with Bootstrap 95% confidence intervals.

	1	2	3	4	5	6	7	8
1.MHC time								
2.Reakiro time	.251^*^	--						
95% CI^a^	[.052,.424]							
3.SSI	-.246^*^	-.029	--					
95% CI	[-.442, -.013]	[-.244, .174]						
4.DES	.117	.191	-.254^*^	--				
95% CI	[-.100, .321]	[-.007, .381]	[-.457, -.038]					
5.HHI	.148	.209	-.261^*^	.665^**^	--			
95% CI	[-.060, .354]	[-.009, .406]	[-.448, -.053]	[.535, .764]				
6.MILM	.095	.134	-.334^**^	.415^**^	.558^**^	--		
95% CI	[-.111, .291]	[-.071, .337]	[-.510, -.135]	[.205, .592]	[.381, .685]			
MILM-E	.069	.114	-.226^*^	.597^**^	.688^**^	.747^**^	-.295^**^	-.561^**^
95% CI	[-.128, .265]	[-.094, .326]	[-.421, -.014]	[.439, .712]	[.556, .785]	[.622, .831]	[-.485, -.088]	[-.696, -.390]
MILM-R	.094	.098	-.304^**^	.196	.300^**^	.888^**^	-.060	-.111
95% CI	[-.126, .287]	[-.090, .305]	[-.482, -.115]	[-.034, .405]	[.111, .462]	[.836, .920]	[-.265, .155]	[-.299, .096]
7.ECQ	.023	.035	.229^*^	-.401^**^	-.380^**^	-.179	--	
95% CI	[-.169, .222]	[-.168, .238]	[.025, .425]	[-.563, -.210]	[-.556, -.185]	[-.379, .019]		
8.OQ-45	-.093	-.075	.356^**^	-.602^**^	-.587^**^	-.343^**^	.483^**^	--
95% CI	[-.290, .123]	[-.273, .149]	[.156, .535]	[-.724, -.456]	[-.711, -.431]	[-.514, -.138]	[.315, .636]	

** indicates *p* < .01. * indicates *p* < .05 (Benjamini Hochberg corrected).

^a^Bootstrap results are displayed below each correlation coefficient and are based on 1000 bootstrap samples.

## Discussion

This quantitative study corroborates the results of previous qualitative studies on differential expressions of persistent death wishes and empirically elucidates the severe suffering of persons with SPMI and a persistent death wish [16,17]. They are long-term enrolled in mental healthcare, sometimes for decennia, concerning treatment for various and often comorbid psychiatric vulnerabilities. The severity of the suffering is high, compared with 20 other psychiatric and non-clinical samples worldwide. The Reakiro sample of persons with SPMI and a persistent death wish almost exclusively scores worse on all measured protective and risk factors concerning suicide. They report very strong suicidal ideation and psychosocial dysfunctioning, elevated existential anxiety and a lowered experience of hope, meaning and empowerment, compared with other samples. Almost all participants scored well above the clinical cut-off for psychosocial dysfunctioning, compared to a Dutch inpatient sample [57]. Approximately a quarter was actively engaged in the formal PE procedure. The majority reported feeling unchanged in their life or death wish, indicating chronicity and feeling stuck in their dilemma.

However interestingly, a small minority (5.6%) reported to not hold a current death wish and 16 persons reported lower to even absent suicidal ideation. Indeed, even some individuals reported a wish to live and another subgroup showed possible ambivalence by indicating to simultaneously hold a wish to live and a wish to die. Others related more passively to life and death by indicating a neutrality towards both wishes. The results show a wide, complex spectrum of relation towards life and death. It seems that the relationship between the wish to live and the wish to die is more orthogonal in nature, meaning that both wishes are constructs in itself which form a relationship rather than mirror images of a one-dimensional spectrum. Not everyone who wishes to die, does not wish to live (and vice versa). These results corroborate the differentiated spectrum of death wishes, found in qualitative studies on PE and suicidality [16,18]. A simplified, dichotomous approach to life and death should probably be abandoned, because it is not attuned to the complex experiences of persons with SPMI and a persistent death wish.

How did users evaluate the Reakiro care? Reakiro users seemed to perceive a small strengthening of the choice for life and almost no weakening of the choice for death. All Reakiro care modalities were on average appraised as helpful, with individual counselling services feeling as the most helpful part of Reakiro care. Users also indicated that Reakiro offered an added value to their care that they did not find in their other mental healthcare services. Although we cannot draw any strong or causal conclusions, the combination of these findings seems to suggest that the Reakiro care appeals to a significant subgroup of persons with SPMI and a persistent death wish. No key factors were found to be significantly associated with time enrolled in Reakiro care. Minor positive trends were found with empowerment, meaning and hope. This makes sense as 58.9% of the sample had only just started in Reakiro (≤2 months of enrolment) and might thus show more change over time. This sample is part of a cohort followed for a year in Reakiro, so these cross-sectional data might reflect a baseline and forthcoming longitudinal data might shed more light on actual change processes. Another possible explanation is that non-response or poor response is very common in persons with SPMI and therefore to be expected [59]. Either way, due to the cross-sectional design limits we can only speculate on what it is exactly that persons find in Reakiro. Why do persons even enrol in Reakiro, if their hopes are so low? An in-depth interview study with 10 participants in Reakiro highlights that acknowledgement of the persisting death wish and of the suffering is key to allow small, brittle reconnections to emerge with themselves, others, and ultimately with life [19]. Existential, palliative and recovery-oriented practices offer the potential to enhance contemporary care for this target population [15].

## Clinical implications

An open, phenomenological, inquisitive, explorative, humble and non-judgemental stance towards a persistent death wish would probably be the most suitable as this quantitative study confirms previous qualitative results displaying the complexity and heterogeneity of this personal and deep experience of a persistent death wish [60]. Maybe caregivers sometimes resign to a more simplistic apprehension of life and death to soothe their own existential anxieties? [61] It can be very challenging and disruptive for the caregiver to immerse oneself in the persistent death wish of the person. Careful existential, empathic attunement and a therapeutic presence with and for the person with a persistent death wish are proposed as necessary skills to navigate these experiences [60,62].

Hope, empowerment and experienced meaning seem to form a conglomerate of protective factors, while existential anxiety, psychosocial dysfunctioning and suicidal ideation are once again confirmed to be risk factors for suicide, in accordance with suicidology and PE literature. Future healthcare can focus on bolstering these positive experiential processes and thereby soothing existential anxiety and psychosocial dysfunctioning. A new ground to embark on is how suicide prevention can be integrated with palliative psychiatry to form an attuned care for persons with a persistent death wish [63]. It is an interesting finding to see that time spent in mental healthcare is associated with less suicidal ideation, but time spent in Reakiro is not. Could this possibly mean that there is a different relational stance towards suicide? Reakiro caregivers use deliberate risk management (after careful team consideration) by not coercing into safety (e.g., isolation cells), but increasing the frequency of the therapeutic relationship and empowering shared decision making towards possible suicide risk with collaboration with both the full care network and the person [64]. This allows the person to fully experience their ambivalence and own their life and death dilemma and explore it to the bottom.

## Limitations and future research

A limitation of the study is that it did not address the distinction between acute and chronic suicidality. At the moment of designing this study, no questionnaires were found that assessed one or both subtypes of suicidality. Interestingly, a promising study reports on the development and validation of a new scale to measure chronic suicidal ideation: the Chronic Suicidal Ideation Inventory-5 (CSI-5) [7]. Clinical practice could benefit from future research disentangling the suicidal subgroups and exploring the possible differences in care needs of these subgroups. Another limitation is that the psychometrically validated questionnaires were not validated in the Belgian context. The Reakiro questionnaires used are unvalidated instruments, which limits reliability and warrants caution in drawing conclusions.

The design of the study also limits the conclusions that can be drawn, as causality could not be researched. The question whether Reakiro had any impact on choice for life or death could have been subject to bias, especially because some persons received this question after intake and others were already enrolled for a longer time period in Reakiro. A control group would not be ethically justifiable, so no direct comparisons could be made to persons with similar trajectories outside of Reakiro. The comparisons with closely related populations (inpatient samples of persons with mood, personality, and psychotic disorders) have merit, but are of course not a direct representative match with our specific population of persons with SPMI and a persisting death wish.

The aim was to explore and clarify the population and its characteristics and assess how the users evaluated the Reakiro care. A future Reakiro study will use longitudinal data to shed more light on how these persons change over the course of one year in their life and death struggle and in the underlying risk and protective factors. Future studies could research if palliative psychiatric models like Reakiro are associated with reduced morbidity rates in this population. Longitudinal designs are crucial to capture experiential change processes over time and could possibly lead to more robust empirical models that underpin good clinical practices for this target population.
